# Anti-VEGF therapy - a new hope in AMD treatment

**DOI:** 10.22336/rjo.2025.52

**Published:** 2025

**Authors:** Ana-Maria Stoica, Sanda Jurja, Nejla Dervis

**Affiliations:** 1 Department of Ophthalmology, “Sf. Apostol Andrei” County Emergency Clinical Hospital, Constanţa, Romania; 2 Department of Ophthalmology, General Medicine Faculty, Ovidius University, Constanţa, Romania; 3 Department of Diabetes, “Sf. Apostol Andrei” County Emergency Clinical Hospital, Constanţa, Romania

**Keywords:** neovascular AMD, anti-VEGF, retina, gene therapy, AMD = age-related macular degeneration, CNV = choroidal neovascularization, RPE = the retinal pigment epithelium, PEDF = pigment epithelium–derived factor, VEGF = vascular endothelial growth factor, PIGF = placental growth factor, BCVA = best-corrected visual acuity, PDT = photodynamic therapy, DARPins = designed ankyrin repeat proteins

## Abstract

Choroidal neovascularization (CNV) secondary to age-related macular degeneration (AMD) represents a significant global cause of visual impairment. Contemporary therapeutic approaches for neovascular AMD focus on inducing regression of CNV by inhibiting critical growth factors involved in angiogenesis. For nearly two decades, vascular endothelial growth factor (VEGF) has been the principal therapeutic target, with multiple intravitreally administered agents developed to achieve anatomical restoration and improved visual outcomes through sustained dosing.

## Introduction

Age-related macular degeneration (AMD) is the leading cause of severe vision loss in individuals aged 65 years or older in industrialized nations. This bilateral and progressive condition demonstrates notable variability among patients in terms of the rate of visual decline. AMD is an acquired retinal degenerative disease that produces significant central vision impairment through both non-neovascular and neovascular pathways [1].

The global incidence of AMD is expected to increase in the coming decades, emphasizing the pressing need to clarify its underlying pathophysiology and to identify effective therapeutic interventions to prevent vision loss [2].

Because AMD severely compromises central vision—essential for daily activities such as reading and driving—the disease progresses through several stages: early, intermediate, and advanced. Advanced AMD presents as either neovascular AMD (nAMD), defined by the development of choroidal neovascularization (CNV), or as geographic atrophy in the dry form [3]. Persistent CNV activity may evolve into fibrosis and central scarring, permanently damaging the photoreceptors and the retinal pigment epithelium (RPE), ultimately leading to irreversible vision loss [4].

In nAMD, CNV originates from the choriocapillaris. It proliferates through defects in Bruch’s membrane, a process thought to result from an imbalance between vascular endothelial growth factor (VEGF), which stimulates angiogenesis, and pigment epithelium–derived factor (PEDF), which inhibits vascular growth. [5] Three major growth patterns have been described: sub-RPE (type 1), subretinal (type 2), and a mixed form. Type 1 is the most prevalent, whereas type 2, though less common in AMD, tends to occur in younger patients with myopia or chorioretinitis. Initial vision loss in CNV arises from leakage of serum and blood into the subretinal space, retina (macular edema), and beneath the RPE. Such vision loss, due to fluid accumulation, may be reversible, but persistent leakage eventually results in photoreceptor and RPE damage, disciform scarring, and permanent vision loss [6,7].

Vascular endothelial growth factor (VEGF) is a potent endothelial-specific mitogen that promotes angiogenesis and vascular hyperpermeability, particularly under hypoxic conditions [8]. Experimental studies have linked VEGF to the development of retinal and choroidal neovascularization, with elevated VEGF expression observed in the RPE and vitreous of eyes affected by both neovascular and non-neovascular AMD [9-11].

Over the last two decades, considerable progress has been achieved in understanding angiogenesis—the process of new vessel formation in adults [12]. Angiogenesis is triggered by stimuli such as hypoxia, which upregulates growth factors, resulting in vasodilation, increased vascular permeability, and the formation of complex vascular networks. Each step of this process is tightly regulated by the balance between pro- and anti-angiogenic mediators, among which VEGF-A plays the most significant role, alongside others such as fibroblast growth factor, platelet-derived growth factor, and erythropoietin [13].

VEGF-A, first isolated in 1989 by Ferrara and colleagues, is a glycoprotein synthesized in response to hypoxia. Oxygen-deficient cells release hypoxia-inducible factor, which stimulates VEGF-A production and secretion. Extracellular VEGF-A binds endothelial receptors, increasing vascular permeability and promoting endothelial proliferation [14,15]. VEGF-A belongs to a broader gene family that includes VEGF-B, VEGF-C, VEGF-D, VEGF-E, and placental growth factor (PlGF). Four major VEGF-A isoforms arise from alternative splicing: VEGF121 and VEGF165, which diffuse freely, and VEGF189 and VEGF206, which are largely sequestered in the extracellular matrix [1,6].

The current gold-standard treatment for nAMD is intravitreal anti-VEGF therapy [17]. Patients typically undergo injections and monitoring every one to three months, depending on clinical response. However, burdensome, fixed-schedule treatments consistently produce better visual outcomes than irregular dosing regimens. Nevertheless, even under optimal treatment protocols, some patients continue to experience visual decline due to the progression of atrophy or subretinal fibrosis [18].

Anti-VEGF therapies have dramatically altered the management of nAMD, with clinical trials demonstrating significant improvements in best-corrected visual acuity (BCVA) compared with sham injections or photodynamic therapy [19]. Despite these advances, the requirement for frequent injections imposes substantial challenges in terms of both patient adherence and cost, owing to the drugs’ limited half-life and the chronic nature of the disease [17].

Currently approved anti-VEGF agents include ranibizumab (Lucentis), aflibercept (Eylea), brolucizumab (Beovu), bevacizumab (Avastin, off-label), and pegaptanib sodium (Macugen). Multiple studies confirm their ability to produce both visual and anatomical improvements in patients with neovascular age-related macular degeneration (nAMD) [14].

## Current anti-VEGF agents

### Pegaptanib (Macugen)

Pegaptanib is an RNA aptamer that selectively binds human VEGF165. In the VISION clinical trial, patients with subfoveal CNV received pegaptanib or sham injections every six weeks for 48 weeks. A visual improvement of three or more lines was achieved in 6% of treated patients compared to 2% of controls after one year. Pegaptanib gained FDA approval for nAMD; however, due to its limited efficacy and ongoing vision loss in some patients, its use has declined in favor of more effective, newer anti-VEGF agents [17].

### Ranibizumab (Lucentis)

Ranibizumab is a recombinant, humanized antibody fragment targeting all VEGF-A isoforms, thereby blocking VEGF-receptor interactions. Its design includes a murine antigen-binding component and a humanized component, which minimizes antigenicity. Developed by modifying trastuzumab, ranibizumab has structural similarities to bevacizumab. Preclinical data suggested that full-size antibodies may not penetrate the retina efficiently, prompting the development of this smaller antibody fragment. Ranibizumab’s VEGF-binding affinity is approximately 100 times greater than bevacizumab’s. In 2006, the FDA approved ranibizumab for nAMD, following the results of two pivotal Phase III studies: MARINA and ANCHOR [20].

The MARINA trial demonstrated that 95% of ranibizumab-treated eyes maintained or improved vision compared to 62% in the sham group after 12 months, with 40% achieving a gain of 15 letters or more. The ANCHOR trial compared ranibizumab to photodynamic therapy (PDT), revealing superior outcomes with ranibizumab, as 95% of treated eyes maintained or improved their vision compared to 64% with PDT [21,22].

However, real-world evidence has shown more modest visual gains than those observed in controlled trials, highlighting challenges in replicating trial-level adherence and outcomes in practice.

### Bevacizumab (Avastin)

Bevacizumab is a full-length monoclonal antibody with both Fab and Fc fragments, enabling binding to VEGF with two antigen-binding domains, unlike ranibizumab’s single domain. The ABC trial showed that repeated bevacizumab injections improved both functional and structural outcomes in nAMD. Compared with ranibizumab, bevacizumab has a longer systemic half-life, which may potentially prolong its effects. The CATT trial confirmed the non-inferiority of bevacizumab compared with ranibizumab; however, five-year follow-up results revealed that the vision gains achieved at two years were not sustained over time. Notably, more frequent injections were associated with larger areas of geographic atrophy [23].

Five-year results of the CATT study suggested that the vision attained at the end of 2 years was not maintained at 5 years, but at least 50% of the eyes had vision of at least 20/40. It also suggested that more frequent anti-VEGF injections resulted in increased area of geographic atrophy. It confirmed the role of anti-VEGF therapy as the mainstay in treatment for neovascular AMD. Safety analyses from CATT indicated slightly higher rates of systemic adverse events with bevacizumab (24.1% vs. 19.0%, P = 0.04). However, rates of serious vascular events, such as arteriothrombotic events (non-fatal strokes, myocardial infarctions, and vascular death), venous thrombotic events, and mortality were similar between groups [17].

Other large trials, including IVAN, MANTA, and GEFAL, consistently demonstrated the non-inferiority of bevacizumab compared to ranibizumab across various treatment regimens, supporting its role as a cost-effective alternative.

### Aflibercept (Eylea)

Aflibercept is a recombinant fusion protein composed of domains from human VEGF receptors 1 and 2, enabling it to bind VEGF-A, VEGF-B, and placental growth factor with high affinity.

Clinical trials enrolling approximately 2,400 patients with neovascular AMD demonstrated that aflibercept was non-inferior to monthly ranibizumab. In these pivotal studies, aflibercept was administered at either 0.5 mg every four weeks, 2 mg every four weeks, or 2 mg every eight weeks, following three initial monthly doses. In contrast, ranibizumab was administered every four weeks. The primary endpoint was vision maintenance, defined as a loss of fewer than 15 letters on a standardized eye chart after one year of treatment. Across all dosing regimens, Eylea was shown to be as effective as ranibizumab in maintaining vision in patients with wet AMD: the proportions of patients who maintained vision were 96.1% (517 out of 538), 95.4% (533 out of 559) and 95.3% (510 out of 535) for 0.5 mg Eylea every four weeks, 2 mg Eylea every four weeks and 2 mg Eylea every eight weeks, respectively, compared with 94.4% (508 out of 538) of patients treated with ranibizumab every four weeks [2,4].

During the second year, treatment effectiveness was preserved, with many patients successfully managed on extended dosing intervals averaging 10 weeks. Only a minority required more frequent injections, such as monthly dosing. Follow-up studies further supported aflibercept’s durability, confirming its ability to maintain visual outcomes while reducing treatment burden [2,5].

### Brolucizumab (Beovu)

Brolucizumab is a small, single-chain antibody fragment that offers several advantages, including its compact size, superior tissue penetration, rapid systemic clearance, and flexible delivery potential. Reports have indicated the occurrence of occlusive retinal vasculitis (approximately 1%) and intraocular inflammation (around 4%) following intravitreal administration. These adverse events can be managed with corticosteroids (systemic, intravitreal, or topical), although visual prognosis in cases of occlusive vasculitis remains poor.

Beovu was evaluated in two pivotal clinical trials conducted over roughly two years, enrolling about 1,800 patients with neovascular AMD. In these studies, Beovu was administered monthly for the initial three doses, followed by dosing every 8 or 12 weeks, and compared with aflibercept, which was given monthly for three doses and subsequently every 8 weeks. The primary endpoint was the change in visual acuity after one year, assessed by the number of letters recognized on a standardized eye chart. The durability of the treatment effect was also examined in the second year [2,6].

Results demonstrated that Beovu was comparable to aflibercept in maintaining visual function. In the first trial, Beovu-treated patients achieved an average improvement of 6.4 letters compared to 7 letters with aflibercept. In the second trial, the gains were 6.9 letters with Beovu and 7.6 letters with aflibercept. The therapeutic effect of Beovu was generally sustained during the second year of follow-up.

### Faricimab

Faricimab (F. Hoffmann-La Roche Ltd, Basel, Switzerland) is the first bispecific antibody specifically engineered for intraocular application. This innovative humanized bispecific immunoglobulin G monoclonal antibody independently binds to and neutralizes both VEGF-A and angiopoietin-2 (Ang-2). Furthermore, its crystallizable fragment domain was modified to shorten systemic half-life and minimize inflammatory potential. The angiopoietin-tyrosine kinase (Ang/Tie) signaling pathway plays a crucial role in maintaining vascular stability [27].

Ang-1, produced by pericytes, activates the Tie2 receptor on endothelial cells, promoting vascular stability and homeostasis. In contrast, Ang-2, which becomes upregulated under pathological conditions, contributes to inflammation and vascular destabilization, leading to leakage and the formation of new blood vessels (neovascularization). By competing with Ang-1 for Tie2 binding, Ang-2 inhibits Ang-1-mediated receptor activation, destabilizing endothelial cells and increasing their sensitivity to VEGF.

Faricimab has undergone evaluation in several Phase 2 and Phase 3 clinical trials for neovascular AMD. Among these, the TENAYA and LUCERNE Phase 3 randomized, double-masked, multicenter studies compared its efficacy and safety with aflibercept. In both trials, improvements in best-corrected visual acuity (BCVA) with faricimab were non-inferior to those achieved with aflibercept [17].

Adverse event rates were low across both treatment groups, with intraocular inflammation observed in 2.0% of patients treated with faricimab and 1.2% of patients treated with aflibercept. Importantly, no cases of retinal vasculitis or occlusive retinitis were reported in either study.

### Abicipar

Abicipar pegol is part of a novel class of therapeutics known as designed ankyrin repeat proteins (DARPins). These molecules are small, highly stable, and engineered binding proteins that incorporate ankyrin repeat domains. Their design enables an optimized balance of binding affinity, molar concentration, and half-life, ultimately resulting in a prolonged intraocular duration of effect. Abicipar is a pegylated DARPin engineered to bind all isoforms of VEGF-A with greater affinity and specificity than Ranibizumab.

Two global Phase III clinical trials, SEQUOIA (NCT02462486) and CEDAR (NCT02462928), both conducted under identical protocols, recruited more than 1,600 patients with treatment-naïve neovascular AMD. Pooled analyses demonstrated that over 90% of participants across all study arms achieved stable vision. These results confirmed the non-inferiority of abicipar when compared with ranibizumab, while requiring fewer injections (6 or 8 annually versus 13 with ranibizumab). This suggested the potential for significantly reducing treatment burden [17].

Nonetheless, safety concerns emerged regarding intraocular inflammation (IOI). The CEDAR and SEQUOIA studies reported IOI rates of 15.4%, with approximately 1.7% of patients experiencing severe vision loss. The subsequent MAPLE trial, a 28-week open-label study evaluating a modified formulation, demonstrated a reduction in inflammation rates to 8.9%, with severe cases at 1.6%. Although these results indicated progress, the safety profile remained less favorable compared to established agents [26].

Despite these challenges, DARPins remain a promising therapeutic category. Their potential for extended durability and reduced injection frequency addresses a critical unmet need in the management of neovascular AMD. However, further refinement of molecular formulations and additional large-scale clinical trials are essential to establish a favorable risk-benefit profile. When benchmarked against established anti-VEGF agents such as bevacizumab, ranibizumab, and aflibercept, DARPins may ultimately provide a necessary addition to the therapeutic armamentarium, provided safety issues are adequately mitigated.

### KSI-301

KSI-301 is an antibody-biopolymer conjugate designed to extend intraocular drug durability. It consists of a humanized anti-VEGF antibody, structurally similar to ranibizumab, conjugated to a phosphorylcholine-based polymer with an ultra-high molecular weight. This design substantially enhances intraocular stability and prolongs the therapeutic effect. With a molecular weight of 950 kDa, KSI-301 is the largest anti-VEGF molecule currently under investigation. Its clinical dose (5 mg, equivalent to 3.5 µM) corresponds to a molar dose approximately seven times greater than that of ranibizumab (48 kDa, 0.5 µM) [27].

In a Phase IB open-label, randomized clinical study, KSI-301 was evaluated at doses of 2.5 mg and 5.0 mg in patients with neovascular AMD, diabetic macular edema, and retinal vein occlusion. In the neovascular AMD cohort, 55% of patients maintained disease control for six months without retreatment. Following retreatment, 84% were able to extend intervals to four months or longer without requiring additional therapy.

These findings suggest that KSI-301 has the potential to markedly reduce the treatment burden commonly associated with current anti-VEGF agents. Its prolonged durability offers a promising therapeutic advance, although larger, long-term clinical trials are necessary to validate its safety, efficacy, and sustained outcomes.

### Topical treatments

Several topical agents, including tyrosine kinase inhibitors (TKIs) and other small molecules, have been explored to inhibit VEGF and PDGF pathways. Examples include pazopanib, squalamine lactate, regorafenib, and LHA510. However, none demonstrated sufficient clinical efficacy to replace or reduce the need for intravitreal injections, resulting in the discontinuation of their development [14].

## Conclusions

Neovascular AMD can have profound and debilitating consequences on patients’ vision, daily activities, and overall independence. The multifactorial nature of its pathogenesis has driven the development of multiple therapeutic strategies, with anti-VEGF agents serving as the cornerstone by targeting the VEGF pathway. Despite their effectiveness, these agents currently necessitate frequent intravitreal injections, creating challenges for long-term adherence and potentially limiting visual outcomes over time.

Future directions in nAMD therapy focus on enhancing drug durability to reduce treatment frequency and minimize the burden of clinic visits. Strategies include decreasing molecular size, as demonstrated with brolucizumab, to permit higher dosing; modifying binding affinity, as with VEGFR-fusion proteins like aflibercept and conbercept, to slow clearance and extend activity; and developing agents with longer half-lives, such as KSI-301 and abicipar pegol, which have shown promise in sustaining therapeutic effects with extended dosing intervals. Additionally, innovative delivery systems, such as the ranibizumab port delivery system (PDS), may eventually replace repeated intravitreal injections, offering a more continuous and patient-friendly approach.

The advent of anti-VEGF therapies has dramatically transformed outcomes for individuals with wet AMD. The next phase in therapeutic progress will likely hinge on enhancing durability and reducing treatment burden. With numerous ongoing clinical trials and emerging therapeutic modalities, the future landscape for managing this sight-threatening condition remains dynamic and full of potential.
